# A year at the helm

**DOI:** 10.1172/jci.insight.142915

**Published:** 2020-09-03

**Authors:** Kathleen L. Collins

A year has flown by since the University of Michigan–based editorial board began overseeing manuscript adjudication. It’s been my great pleasure to lead this exceptional team of 3 deputy and 17 associate editors, who were carefully selected to provide expertise for the broad range of manuscripts submitted to *JCI Insight* and to reflect the interests of the larger American Society for Clinical Investigation (ASCI) community. Our board of editors also help fulfill ASCI’s commitment to diversity and inclusion, with 9 women and 2 underrepresented minorities. Our weekly editorial board meetings are a lively forum for thoughtful discussion of manuscripts and have been a fantastic opportunity for colleagues across departments to engage with one another.

From the outset, the editorial board has been committed to publishing high-caliber research that advances understanding of disease pathogenesis and treatment, and to improving the experience of authors by providing comments to improve their work rather than a list of reasons why the manuscript should be rejected. We understood that there would be a learning curve to setting our standard for sending papers out for peer review and for publication. However, this year has brought exceptional changes to the entire research community as the COVID-19 pandemic thrust new clinical and scientific challenges to the forefront while grinding research in other areas nearly to a halt.

The need to avoid endless reviewer requests for additional experimentation was made more urgent by the impact of the COVID-19 pandemic that forced the closure of many laboratories. Along with the *JCI* board, we revised the editorial process to address the impact of the COVID-19 pandemic on the research community (1). We empowered the editors to consider overruling reviewer requests if they would not fundamentally change the main conclusions of the manuscript and/or would extend the scope beyond that of a reasonable revision. In some cases, additional time was granted to ensure the authors had time to perform essential revisions.

As laboratories shut down in the spring, we began to see an increase in submission volume to the journal, both of COVID-19–related and –unrelated manuscripts. It is possible that investigators who were relegated to work from home found more time to complete and submit manuscripts, while the unfolding pandemic created an urgent need for information about the virus and clinical course of disease. At the same time, many in our reviewer community have been hard pressed to evaluate manuscripts for a variety of reasons, including increased clinical duties and increased childcare responsibilities. The editorial board continues to have discussions (now via online video meetings) in our evaluation of COVID-19 submissions as we balance the need to disseminate information quickly with the desire to see optimally designed studies. We will continue to evolve in our assessment of papers as the field matures. Perhaps not surprisingly, some of our most highly accessed publications from this last year have been COVID-19 studies. The most highly accessed article describes the presence of neutrophil extracellular traps in COVID-19, which may be an important therapeutic target (2). We have provided a forum for our growing COVID-19 research collection (https://insight.jci.org/posts/171), including an appeal to support the essential requirement for transparency in this time of crisis (3).

To date, our board has handled 1823 manuscript submissions, including 312 transfers from the *JCI*, and we have accepted 482 papers for publication. We are indebted to our reviewers for helping us evaluate these submissions. Along the way, our editorial board has clarified our vision for *JCI Insight* as a forum to publish cutting-edge findings that provide a new way of thinking about a disease — even if the precise molecular mechanism has not been fully elucidated. The *JCI Insight* board finds value in studies that utilize difficult-to-obtain human tissues — especially when they provide new insights into disease processes. We have also become adept at evaluating select *JCI* manuscripts transferred to *JCI Insight* after they were rejected at *JCI*. *JCI Insight* editors are charged with the sometimes-challenging task of crafting a path for modification that would lead to publication in *JCI Insight* based on prior reviews. Our publications have spanned a wide range of disciplines, with the top specialties being immunology, oncology, metabolism, neuroscience, and infectious disease. We have maintained an average time to decision of 28.7 days for manuscripts sent for external review and 5.8 days for papers not sent for review. Our goal is to maintain a speedy process without sacrificing our overarching commitment to rigorous and thoughtful review.

The editorial board has also sought to encourage physician-scientist training through the *JCI Insight* Scholars program. We accepted four exceptionally talented University of Michigan Medical Scientist Training Program (MSTP) fellows who shadow board members during a three-month supervised rotation on the *JCI Insight* board. Their participation helps align the editorial mission of the board with the academic training mission of the faculty board members. It also provides a unique experience for our MSTP fellows. The interest of our board members in physician-scientist training is further reflected by the publication of perspectives on this process, which are among our most highly read articles (4).

We are delighted that the ASCI Council has committed to making *JCI Insight* compliant with Plan S, an initiative from a coalition of funding agencies seeking to ensure research they support is freely available under open access licensing. All articles published in *JCI Insight* have always been freely available online, and now all of the papers in this issue and going forward will be published under a CC-BY license. This move makes *JCI Insight* content fully open access and will make it easier for authors to be compliant with requirements of their funding agencies.

I am exceedingly proud of our new board. Their commitment to rigor, integrity, and careful review are all essential to maintain the high standards of ASCI. I am excited to move into the second year of our tenure working with an exceptional group of people committed to providing a supportive forum for authors to share their work.

## Version 1 09/03/2020

Electronic publication
